# Populations dynamics in Northern Eurasian forests: a long-term perspective from Northeast Asia

**DOI:** 10.1017/ehs.2020.11

**Published:** 2020-05-21

**Authors:** Junzo Uchiyama, J. Christopher Gillam, Alexander Savelyev, Chao Ning

**Affiliations:** 1 The Sainsbury Institute for the Study of Japanese Arts and Cultures, University of East Anglia, 64 The Close, Norwich NR1 4DH, UK; 2 Center for Cultural Resource Studies, Kanazawa University, Kakuma-machi, Kanazawa-shi, 920-1192, Japan; 3 Department of Sociology, Criminology and Anthropology, Winthrop University, 319 Kinard Hall, Rock Hill, SC 29733, USA; 4 Max Planck Institute for the Science of Human History, 07745 Jena, Germany; 5 Institute of Linguistics, Russian Academy of Sciences, Bolshoy Kislovsky Pereulok 1/1, 125009 Moscow, Russia

**Keywords:** Northern Eurasian Greenbelt, migration, Neolithization, Turkic language family, mtDNA

## Abstract

The ‘Northern Eurasian Greenbelt’ (NEG) is the northern forest zone stretching from the Japanese Archipelago to Northern Europe. The NEG has created highly productive biomes for humanity to exploit since the end of the Pleistocene. This research explores how the ecological conditions in northern Eurasia contributed to and affected human migrations and cultural trajectories by synthesizing the complimentary viewpoints of environmental archaeology, Geographic Information Science (GIS), genetics and linguistics. First, the environmental archaeology perspective raises the possibility that the NEG functioned as a vessel fostering people to develop diverse cultures and engage in extensive cross-cultural exchanges. Second, geographical analysis of genomic data on mitochondrial DNA using GIS reveals the high probability that population dynamics in the southeastern NEG promoted the peopling of the Americas at the end of the Pleistocene. Finally, a linguistic examination of environmental- and landscape-related vocabulary of the proto-Turkic language groups enables the outline of their original cultural landscape and natural conditions, demonstrating significant cultural spheres, i.e. from southern Siberia to eastern Inner Mongolia during Neolithization. All of these results combine to suggest that the ecological complex in the southern edge of the NEG in northeast Asia played a significant role in peopling across the continents during prehistory.

## Graphical abstract

**Figure:**
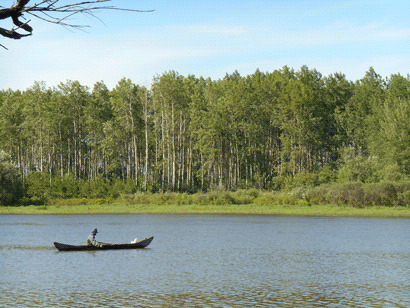

**Media summary:** Northern forests have been an active arena promoting human interaction, exchange and migration across and beyond Eurasia since prehistory.

## Introduction: Northern Eurasian Greenbelt

**Figure 1.**
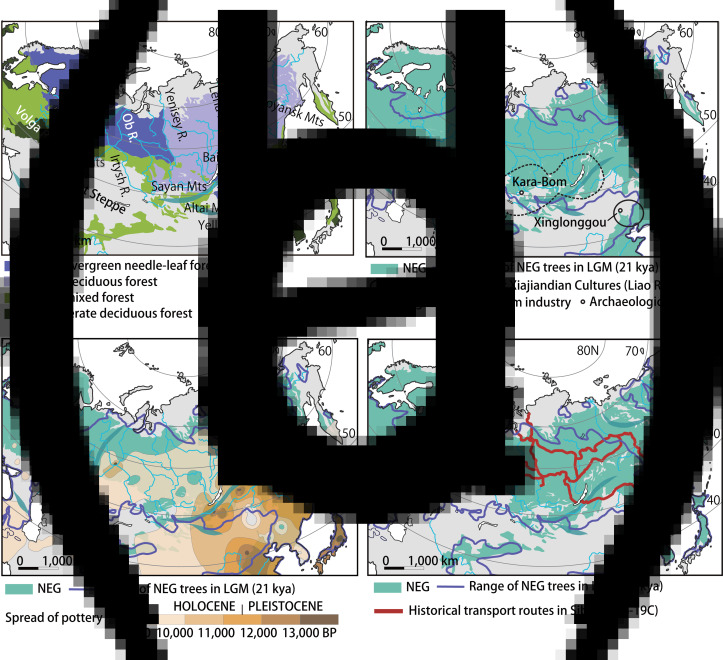
The NEG and human activities from the Upper Palaeolithic to the historical periods. The potential natural vegetation of the present NEG is shown in (a), based on Binney *et al*. (2017, fig. 2). In (b), the potential range of the NEG trees during the LGM is indicated by combining the zones of boreal deciduous, boreal/montane evergreen tree and temperate deciduous trees in fig. 5 of Allen *et al*. (2010). The area of the Kara–Bom industry (ca. 40,000 BP) is suggested by Orimo (2002, figure 6). (c) This part superimposes the NEG, the range of NEG trees in the LGM and the interpolated isochron surface fitted to a subset of radiocarbon dates from sites with early pottery which indicate that early pottery emerged first in East Asia and spread westward across Eurasia through the southern fringe of the NEG. The early pottery map is created based on Gibbs and Jordan (2013, figure 4). Historical transport routes (sixteenth to nineteenth centuries) shown in (d) are reconstructed based on Forsyth (1992, pp. 28–83), map 4 and map 5 and Katsuragawa (1794, pp. 68–69).

The northern forest zone in Eurasia, hereafter the Northern Eurasian Greenbelt (NEG), is one of the largest biomes on Earth. It is a continuum of varied forest types including boreal forests (taiga), cool mixed forests and temperate deciduous forests. Today, the NEG extends from roughly 50° to 70° north latitude, stretching from Northern Europe, just north of the Alps, through Siberia to the Russian Far East of Northeast Asia. Additionally, in East Asia the NEG continues far to the south, down to the lower Yellow River Basin in China and Northern Japan, i.e. approximately 35° north latitude where it transitions to subtropical evergreen forests (Figure 1a). Similarly, simulation of palaeo-vegetation in Northern Eurasia using the LPJ-GUESS dynamic vegetation model (Allen *et al*., 2010), which estimates past vegetation based on annual net primary productivity of a series of plant types, indicates that trees comprising the Holocene NEG may have been widely distributed in Northern Eurasia during the Pleistocene, by ca. 42,000 BP. While forest stands were perhaps widely scattered in the vast grasslands of the northern latitudes, woodland patches were probably larger in the south. Overall, forests were probably distributed throughout the Pleistocene NEG, even during the Last Glacial Maximum (LGM, ca. 25,000–16,000 BP), as important landscape components of the southern Mammoth Steppe (Nogués-Bravo *et al*., 2008; Zimov *et al*., 2012) (Figure 1b). As global warming in the early Holocene greatly increased the number of temperate trees in the lower latitudes and displaced the Mammoth Steppe in the north, forests became thicker and expanded, probably reaching its current form by ca. 10,000 BP.

Genetic and archaeological data suggests that the first groups of modern humans (*Homo sapiens*) reached as far as 50° north latitude by ca. 45,000 BP in Central Asia and ca. 43,000 BP in Eastern Europe. People in Central Asia then went eastwards, spreading in Northeast Asia by ca. 40,000 BP (Bae *et al*., 2017) after having reached the tributaries of the Selenge River in northern Mongolia by ca. 45,000 BP (Zwyns *et al.*, 2019). In Europe, recent re-examination of a human maxilla in the Kent's Cavern site in Devon, UK indicates that anatomically modern humans arrived in Northwest Europe as late as ca. 42,000 BP (Higham *et al*., 2011). These facts strongly suggest that humans encountered northern forests distributed in the southern fringe of the Mammoth Steppe grassland upon their arrival in Northern Eurasia. Recent archaeogenetic studies also documented a long-term and long-distance genetic exchange between populations within the NEG. Both the 24,000-year-old human remains (MA1) associated with the Mal'ta-Buret’ culture, west of Lake Baikal, and the 31,000-year-old individual from the northeastern Siberia genetically fall into the western Eurasian gene pool although it is geographically located in eastern Eurasia (Raghavan *et al.*, 2014; Sikora *et al.* 2019). Another late Upper Palaeolithic individual (Afontova Gora 3) from the west bank of the Yenisei River shared a similar genetic profile with MA1, both of which were collectively termed Ancient North Eurasians (ANE). The ANE genetic profile contributed in different proportions to later populations, such as the Mesolithic foragers from the Karelia and Arkhangelsk regions of Russia (Mittnik *et al.*, 2018) and the ancestors of Native Americans (Raghavan *et al.*, 2014), and is ubiquitous in present-day populations of the NEG (Jeong *et al.*, 2019). Therefore, the NEG is a key area to understand the dispersal of human groups far beyond its bounds, i.e. migration into North America. Consequently, it is crucial to understand the role of the NEG in human history, examining how people have adapted to and interacted with each other in the northern forest environments.

## Methodology: synthesizing environmental archaeology, genetic-geography and linguistics

This paper examines the cultural dynamics of societies in the NEG from the Late Pleistocene forward by an interdisciplinary approach, combining environmental archaeology, genetic geography and linguistics. First, an environmental archaeological perspective establishes the general socio-ecological conditions of the NEG from a long-term perspective, posing a hypothesis about the role of the NEG throughout its cultural history. Next, we cast a spotlight on two major historical events that impacted human history and in which the socio-cultural conditions of the NEG were deeply involved, i.e. the migration of humanity into the Americas during Late Pleistocene and the subsequent spread of the Turkic language family in the Holocene. Through close observation of these events, we estimate to what extent the proposed hypothesis is justified by environmental archaeology. Finally, we synthesize these research observations and propose a new historical perspective based on the cultural–historical roles of the NEG, showing how the NEG has contributed to population dynamics in Eurasia and beyond throughout history.

As each event requires different data and analytical approaches, we adopt different methodologies accordingly. First, we use genetic geography for modelling human migration into the Americas. Using genetic data distributed in both Eurasia and North America, the major migration routes to the Americas and driving forces fostering such population dynamics are demonstrated. Secondly, linguistics highlights the place of origin of the Turkic language family and its early spread across Eurasia by analysing environmental and landscape-related vocabulary and using related research outcomes. The methodology and results arising from these investigations are further detailed within each section.

## Environmental archaeology: socio-ecological background of the NEG

### Long hunting–gathering traditions

Some of the most notable characteristics of human history in the NEG are the long-standing traditions of hunting–gathering economies. The initial migration into the NEG by modern humans may have been conducted by nomadic hunter–gatherers from the west. The Kara–Bom site in the Altai Republic, Russia, which dates back ca. 43,000 BP is an early example (Derevianko and Rybin, 2003; Belousova *et al*., 2018; Figure 1b). Located on the ecological border between the southern forest and the northern steppe, the early Upper Palaeolithic lithic industry at Kara–Bom comprises a variety of blades, showing synchronic similarities to Middle East and Central Europe (Derevianko and Rybin, 2003, pp. 32–33). This industry covers a wide range of the southern NEG from Mongolia to Lake Baikal, which suggests that human migration was carried out through this route (Orimo, 2002; Zwyns *et al.*, 2019).

A wide range of cultural contact via the NEG can also be traced back to the late Upper Palaeolithic. The pressure blade technique, which is one of the major technological innovations for humans, enabling efficient production of lithic blades and micro-blades, appeared in Northeast Asia in the southern part of the NEG around 20,000 BP during the LGM. Further innovation of blade production by pressure flaking from conical cores spread from Southern Siberia, from the Ural Mountains to Central Asia, in the early Holocene (e.g. Brunet, 2012). Damlien (2016) argues that these techniques spread over the East European Plain by ca. 11,200 BP during the transition from the Pleistocene to the Holocene. They subsequently reached southern Norway by ca. 10,300 BP, corresponding to drastic environmental changes at that time. Damlien concludes that this is the result of direct and repeated cultural contact between different traditions, which were common throughout the NEG.

Ceramic vessels appeared among the Upper Palaeolithic hunter–gatherers of the Northeast Asian NEG in the latest stage of the LGM. That is, the dates of the earliest earthenwares are ca. 17,000–14,000 BP in northern Honshu, Japan, and ca. 16,000–15,000 BP in the Amur Basin of the Russian Far East, followed by Lake Baikal and the Altai Mountain regions by ca. 14,000–13,000 BP (Gibbs and Jordan, 2013, table 1). Gibbs and Jordan show an interpolated isochron surface fitted to a subset of radiocarbon dates from the sites with early pottery (Gibbs and Jordan, 2013, figure 4) and suggest that pottery emerged in East Asia and spread westward across the southern NEG and neighbouring regions (Figure 1c). The ‘wave’ of earthenwares from the east reached the Volga Valley by ca. 8000 BP and finally spread into the Baltic Sea regions, arriving in Denmark (i.e. Ertebølle culture) by ca. 7000 BP without being accompanied by an agriculture-based economy (Piezonka, 2012).

Although ceramics may indicate that longer-term settlements and increased territoriality were emerging amongst these increasingly complex hunter–gatherers, truly sedentary lifestyles came much later than the development and use of ceramic wares, probably in conjunction with the Holocene climatic optimum (ca. 7000–5000 BP). In northern Japan, the increase in both shell midden sites and large settlements indicates the spread of a sedentary lifestyle from the middle Initial and Early Phases of the Jōmon period (ca. 9500–5500 BP) onward (Uchiyama, 2017, pp. 141–142). Although archaeological evidence is still quite scarce in the continental part of the NEG, large settlements accompanied by a cultural complex consisting of polished adzes, ceramic vessels and dwelling pits indicate that a sedentary lifestyle appeared in the Rudnaia culture (ca. 8000–5500 BP) in Primorye, Russian Far East (Zhushchikhovskaya, 2006), the Early Chulmun period (ca. 8000–5500 BP) in Korea (Kim *et al*., 2011, pp. 97–107) and the Xinglongwa Culture (ca. 8200–7400 BP) in the Liao River Basin in north-eastern China (Chifeng International Collaborative Archaeological Research Project, 2011). These suggest that more sedentary, complex hunting–gathering gradually spread in accordance with the warming of the climatic optimum, particularly among societies along waterfronts, such as river basins and coastal areas, a pattern that we later argue has its roots in the late Pleistocene.

Farming traditions in the NEG are limited to the southern mixed and temperate deciduous forests, whether they were diffused from urban-irrigation agricultural core areas in the south, such as Central China and the Middle East, or originated in and around the NEG itself, as millet agriculture in the Xinglongwa culture suggests. However, unlike the southern agricultural cores, the acceptance of agriculture as an economic base occurred in a more ‘reluctant’ way. Although complex hunter–gatherers in the NEG already had long traditions of ceramic vessels, they were reluctant to adopt agricultural practices simply because there was no imminent need. That is, even if cereal cultivation started or agriculture expanded into an immediate vicinity, it still took a long time until agriculture became a dominant part of the economy. For example, a recent study has revealed that the domesticated type of broomcorn (*Panicum miliaceum*) and foxtail (*Setaria italica*) millet first appeared in the Lower Liao River and the Lower Yellow River reaches in the southern fringe of the NEG around 8100–7700 BP. In the Liao River, C^14^ dates for broomcorn millet at the Xinglonggou of the Xinglongwa culture are ca. 7700 BP (Leipe *et al*. 2019; Figure 1b). However, while a millet farming-based economy was established in the Middle Yellow River Valley during the Peiligang culture of the Early Neolithic ca. 7000–5000 BP (Liu, 2004, pp. 24–25), millet cultivation did not become an economic base until the Lower Xiajiandian culture (ca. 4200–3600 BP; Figure 1b) in Northeastern China (Shelach, 1999, pp. 119–120). In the Jōmon period of Japan, cultivation of soybean (*Glycine max*) and adzuki bean (*Vigna angularis*) began in Central Honshu, whereas barnyard millet (*Echinochloa esculenta*) was probably domesticated in Northern Honshu in the Middle Jōmon Phase (ca. 5500–4500 BP; Nakayama, 2010, pp. 198–199). Even so, early cultivation in Jōmon Japan was probably carried out only as a part of broad-spectrum subsistence within these complex hunting–gathering societies (Uchiyama, 2017, pp. 143–144). Agriculture-based economies did not start until the Jōmon culture came to an end and the subsequent Bronze–Iron Age Yayoi culture began, when the wet-rice paddy system was introduced (ca. 3000–2300 BP), originating in the Chinese Yangtze River Basin of the southern temperate evergreen forest zone (Uchiyama, 2017, pp. 146–147). In the western part of the NEG along the Baltic Sea, coastal hunting–gathering societies with pottery persisted until immigrants speaking the Indo-European languages of the Corded Ware culture arrived and spread from the Great Steppe (ca. 4900–4300 BP), even though agricultural economies based upon cereals and livestock were well established in adjacent regions from the Balkan Peninsula to Germany (ca. 8000–4500 BP, represented by the Starčevo and the LBK cultures) (Piezonka, 2012; Haak *et al*., 2015).

It is noteworthy that the long-standing traditions of the hunting–gathering economies of the NEG are not necessarily a consequence of the comparatively harsh, cold climate. On the contrary, cultures in the NEG were very innovative and active with far-reaching cultural exchange across the continent throughout prehistory, as seen in the spread of both the pressure technique of lithic tools and early pottery before the Holocene. While agriculture-based economies were introduced from the adjacent steppe or temperate evergreen regions around 6000–3000 BP in the NEG, such social change was rather limited to the southern NEG where the climate allowed farming, and there was a long, ‘reluctant’ period before its adoption as an economic base. Over large parts of the NEG, excluding Northeast China and Eastern Europe, hunting–gathering remained significant factors in socio-economic systems until historical times, although reindeer herding appeared by the first millennium BCE and reindeer pastoralism became growingly important in the taiga of the northern NEG and the Arctic tundra (e.g. Vainshtein, 1980).

Now the questions that remain are: why was the hunting and gathering-based economy of the NEG persistent for such a long time period in history? What driving forces made these cultures innovative and active across the continent? And, how did the NEG contribute to the migration of human groups on Earth?

### Environmental setting

To answer these questions, it helps to first consider the environmental characteristics of the NEG which influenced socio-economic activities, i.e. potential resources and geographical features.

There have been abundant wild resources in the woodlands of the NEG since the Late Pleistocene. At that time, the NEG was already rich in game as it stood on the ecological boundary between the northern Mammoth Steppe and the southern Temperate Forests, where terrestrial mammal groups from these different biomes met. For example, in Central Honshu of Japan there were two major terrestrial mammal groups, the mammoth fauna in the taiga and cold grasslands and the Palaeoloxodon–Sinomegaceroides complex, represented by Naumann's elephant (*Palaeoloxodon naumanni*) and giant deer (*Sinomegaceros yabei*), that were adapted to the more temperate forests (Iwase *et al*., 2012). After the LGM and as the NEG expanded, animals adapted to increasingly temperate forests replaced the Pleistocene megafauna. While coniferous forests are predominant in taiga, the northern NEG today offers habitats to numerous mammals, such as brown bear, wolf, elk, moose, wisent, forest reindeer, lynx and small fur-bearing species like sable, stoat, ermine, polecat and squirrels. The southern NEG is a large transitional band along the Great Steppe, as coniferous trees are gradually replaced by temperate ones. Consequently, species living in both taiga and temperate forests can be seen. Species such as black bear, snow leopard, amur tiger, red deer, sika deer, roe deer and wild boar are added to the animal list (e.g. Velichko, 1995). On the other hand, the NEG is rich in waterways and wetlands, which about 780 species of water birds inhabit, making the NEG one of the world's major summer breeding grounds of migratory birds (Dobrynina and Kharitonov, 2006). Freshwater fish are also abundant in the NEG, including the carp family, such as roach, dace and rudd, and others belonging to orders Salmoniformes, like trout, and Acipenseriformes, such as sterlet and northern pike. Added to these, a variety of maritime resources are available for people living near the coasts of Japan, Okhotsk and the Baltic Seas. Regarding plant resources, nuts and acorns are particularly rich in the temperate deciduous forests in the southern NEG. Abundant wild resources undoubtedly supported fishing–hunting–gathering ways of life.

The NEG is also an area rich in waterways and transport; using these waterways was another important component of the NEG. Historical records attest that interconnected waterways, consisting of major rivers and their tributaries, were used as communication and transit routes from the East European Plain to Russian Far East through Siberia, especially before the Russian Empire constructed roads in the 1730s. Waterways were the best way to travel in Northern Eurasia to avoid the difficulties of inland routes, often with overgrown plants and seasonal freeze–thaw cycles of overland surfaces. For example, fur trade routes from the west, across Siberia into Northeast Asia, and based upon historical waterways used by indigenous peoples, were exploited as the Russian Empire expanded eastward from the late sixteenth to the early eighteenth centuries (Forsyth, 1992; Figure 1d). Loads were conveyed by boats in summer and sleds in winter when rivers were frozen. Portage overland was implemented mainly to cross between river systems with minimum effort, cost and distance.

River routes and their cross-drainage connections of the NEG made rapid movement of people and goods possible across Northern Eurasia. For instance, Kōdayū Daikokuya, a Japanese merchant in the late eighteenth century, drifted and went to Russia in 1782 and spent 11 years travelling throughout the country. Upon his return to Japan, the government, under the strict seclusion policy, carefully investigated Daikokuya and compiled a survey which includes detailed information about his travels in Siberia. The survey attests to the wide use of riverways for movement in Siberia. For example, it took only one month to travel by boat 2700 km from Irkutsk, near Lake Baikal, to Yakutsk, on the central Lena River, from 20 May to 19 June 1791 (Katsuragawa, 1794, pp. 68–69). Ethnographies of indigenous groups testify that even open seas were not obstacles for them, with both rivers and seas used for trading activities, enabling people to establish strong social bonds between different cultural groups. For example, the Ainu people of Hokkaidō and Sakhalin and the Nivkh and Ulcha groups of the Lower Amur Valley engaged in trade connecting the Qing Dynasty of China and the Tokugawa Shogunate of Japan in the eighteenth and nineteenth centuries. The people in the Amur Valley brought furs to exchange for silk clothes with Chinese merchants, whereas the Ainu obtained rice and iron tools from Japan. Then, local people exchanged these Chinese and Japanese commodities. In this way social groups in the NEG were playing an important role to connect different farming societies to the south (e.g. Sasaki, 1996; Zgusta, 2015).

### Hypothesis: the NEG as a trans-continental cultural system

As discussed, social groups in the NEG have long traditions of hunting–gathering economies. The abundant wild resources, which becomes more diverse in the southern part and maritime coasts, supported forager subsistence, making hunting–gathering substantially more persistent. Likewise, the migration, transport and trade networks based on interconnected riverine ‘highways’ across Northern Eurasia were probably another factor supporting hunter–gatherer societies, by enabling cross-cultural exchange and strengthening of social bonds between different regions. Such high-level, cross-cultural exchange may have been the primary source of creating cultural innovation, as seen in blade production by pressure flaking from conical cores and the development of ceramic technology for cooking and long-term storage.

These observations generate a further hypothesis about the cultural function of the NEG. That is, despite comprising many societies with differing cultures region-by-region, the NEG may have functioned as a singular cultural network system since prehistory from Northeast Asia to Northern Europe. Additionally, this NEG cultural network may have implications beyond the hunting–gathering world in the northern latitudes. As seen in the dispersal of pottery technology from Northeastern Asia in the early Holocene and the intermediary trading activities between the Qing Dynasty and Japan in pre-modern times, the NEG may have functioned as an intermediate bridge between different societies that reside outside of the NEG and perhaps encouraged cultural diffusion and population movement beyond. Next, let us evaluate these concepts by focusing on two major historical events which the NEG is believed to have been deeply rooted in: the peopling of the Americas and the expansion of the Turkic language family.

## A genetic–geographical perspective

The influence of the NEG on humanity across greater Eurasia is not restricted to the Holocene; it extends well into the Late Pleistocene (ca. 45,000–12,000 BP). While the extent and composition of NEG forests were more southerly and discontinuous during this period (Figure 1b), they nonetheless influenced the cultural trajectories and ebb and flow of humanity throughout northern Eurasia and even beyond, into the Americas. Initial migration and settlement of the NEG in East Asia probably occurred along a northerly route from west to east by ca. 45,000 BP (Zwyns *et al*., 2019). The NEG during the Late Pleistocene probably witnessed a dynamic dance between humanity and nature. That is, during stadial episodes, as the environment cooled, populations moved southward out of the upper northern latitudes (50–70°). Conversely, when warming occurred during interstadials, populations shifted again northward to the opening ecotones above 50° north latitude. During the southerly shifts, northern populations probably mated with other modern humans that had arrived in Asia slightly earlier along the southern coastlines (by ca. 55,000 BP). This episodic pattern of migration and interaction in the Late Pleistocene NEG not only influenced the cultural dynamics and trajectories of the area, but also set the stage for the peopling of the Americas. Genetic evidence suggests that the ancestors of Native Americans share certain mitochondrial DNA (mtDNA) haplogroups (A–D) and genomic substratum with populations in the NEG.

### Cultural, physical and bio-geographical evidence

Prior bio-geographical models have revealed the most likely origins of the migratory populations entering the Americas and the routes they may have taken (cf. Anderson and Gillam, 2000; Banks *et al.*, 2006; Gillam *et al.*, 2007; Gillam, 2007, 2013). The locations found to have the greatest potential as Paleoamerican cultural hearths correlate well with the NEG's LGM (25,000–16,000 BP) geographical range (Figure 1b; see also, Banks *et al.*, 2006; Gillam *et al.*, 2007). The easternmost expansion of humanity prior to 16,000 BP is evidenced in the archaeological record as the Pacific coastlines of Honshu, Hokkaidō and Sakhalin Islands, hereafter the Archipelago, ranging from 142 to 153° north latitude, that were probably inhabited by ca. 38,000 BP (Vasil'ev *et al.*, 2002; Ono *et al.*, 2002; Tsutsumi, 2012). Importantly, the MIS 3 occupation of the Ryukyu Islands evidences advanced ocean-fairing capabilities and coastal adaptations in the region (cf. Kaifu *et al.*, 2015), although the coastal adaptations of the Palaeolithic Archipelago populations remain poorly understood and largely ignored in the literature. As both near- and off-shore watercraft are highly specialized technologies and Palaeolithic stone axes capable of watercraft construction are well documented in the Archipelago's interior, it is probable that significant coastally adapted populations complemented their terrestrial- and riverine-adapted counterparts. These factors all favour the periphery of the NEG along the Archipelago as a cultural hearth during the LGM for coastal migration into the Americas soon thereafter, ca. 18,000–16,000 BP.

### Supporting archaeological evidence

It is proposed that a higher degree of cultural diversity during the LGM of the coastal and adjacent riverine NEG of the Archipelago set the stage for the initial waves of migration to the Americas. That is, there were probably mobile maritime adapted populations along the coast that became increasingly pressured by more sedentary populations of the interior riverine. Similarly, the more mobile populations of the northern latitudes and inland NEG forest and steppes of Siberia probably migrated in a second wave soon thereafter (perhaps within 500–1000 years). Pottery, beginning around 17,000 BP in the Archipelago and Far East, suggests a shift towards increasingly sedentary foragers throughout the interior NEG (Kudo, 2012; Ono *et al*. 2002; Taniguchi 2006). This probably led to regional territoriality that pressured the lifeway of more traditional, highly mobile fisher–hunter–gatherers along the NEG's Pacific Rim, i.e. competition and conflict provided a cultural incentive to migrate northeast along the coast towards the Americas, beyond merely having the ability to do so.

Honshu, Japan's main island, and the (then) mainland attached Sakhalin–Hokkaidō Peninsula of the Archipelago are particularly strong candidates as a Palaeoamerican hearth. Honshu had bifacial point and backed blade technologies from around 22,000 to 16,000 BP (Kudo, 2006; Ono *et al.*, 2002), lending technological correlates to the shared ecological and geographical factors with the Americas, as demonstrated by the models. Likewise, genomic evidence of mitochondrial DNA (mtDNA) haplogroup, D4h3a, and Y-chromosome haplogroup, Q-L54*(xM3), in the Pleistocene Anzick-1 burial in North America and Q-L54*(xM3) in Central and South America point to a Pacific Rim migration from more southerly latitudes of the Far East, as opposed to central Siberia or northerly Beringia, prior to 16,000 BP (Battaglia *et al*., 2013; Rasmussen *et al.*, 2014).

### The genomic evidence

Recent ancient genomic studies add to our understanding of the origins of the ancestors of Native Americans (Sikora *et al*. 2019; Raghavan *et al.*, 2014; Moreno-Mayar *et al.*, 2018). A complex population history is associated with the culture found at the Yana RHS, serving as the first inhabitants of northeast Siberia, west of Beringia. Later, people that harbour East Asian ancestry admixed with the descendants of Yana RHS at about 20–18 kya, forming the Native American lineages and Palaeo-Siberian ancestry related to an individual from the Duvanny Yar site on the Kolyma River. Both of these share greater affinity to the 24,000-year-old Mal'ta individual from the Lake Baikal region than the Yana RHS, suggesting that the admixture forming Native American ancestry could have happened in more southerly latitudes (Sikora *et al*. 2019; Raghavan *et al.*, 2014).

There is a compelling argument for the southerly latitudes of the NEG in Far East Asia, particularly the Archipelago, to be a cultural hearth for the coastal peopling of the Americas based upon bio-geographical and archaeological data and analyses (Banks *et al.*, 2006; Gillam *et al.*, 2007). However, the Archipelago has a particularly complex cultural and migration history, complicated further by the fact that the acidic soils are not favourable for the preservation of ancient DNA (cf. Oota *et al*., 1995) and only very limited ancient DNA studies from the Archipelago are available (Oota *et al*. 1995; McColl *et al.* 2018; Kanzawa-Kiriyama *et al.* 2017). Modern mtDNA and Y-chromosome sequences are mixed with late admixtures from the Russian Far East and Korean Peninsula, consecutively, further complicating matters. More specifically, it is estimated that 65% of mainland Japanese (Honshu) and 20% of Ainu (northern) and Ryukyuan (southern) gene pools are from continental gene flow after the late prehistoric Yayoi Period (post-3000 BP; Horai *et al.*, 1996).

Modern populations in Japan are supposedly best represented by the Dual Structure model of Japanese origins (Matsumoto, 1988; Hanihara, 1991). That is, beginning in the final Late Palaeolithic (post-18,000 BP), migrations occurred on the Archipelago that were the prehistoric Jōmon and historic Ainu progenitors and a later population influx occurred during the Yayoi and Kofun (post-2000 BP) migrations, both via the Korean Peninsula (Omoto and Saitou, 1997). However, because the model was constructed based on morphological analyses of skull remains and the oldest measurable human skull so far obtained in Japan is dated ca. 18,000 BP (Matsu'ura and Kondo, 2011), there is still room for examination regarding Palaeolithic populations that represent over half of the Archipelago's prehistory, between 40,000 and 18,000 BP. The earliest known site is the Ishinomoto site, Kumamoto Prefecture, Kyushu, C^14^ dated from 39,940 to 35,180 BP (Tsutsumi, 2012). During this time period, it is likely that people arrived from the north, originating in the Amur Basin; these populations are most crucial to linking the Archipelago's gene pool to the greater NEG and the problems of the peopling of the Americas.

**Figure 2.**
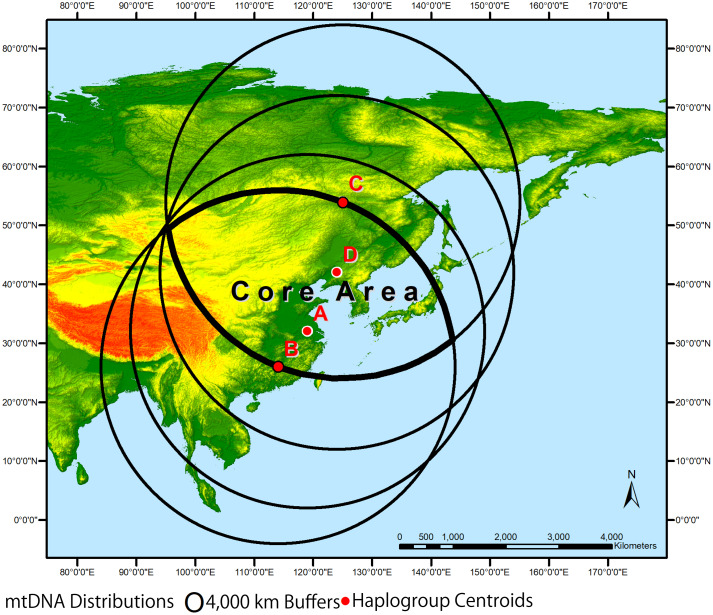
Native America mtDNA haplogroups are known to be dominated by groups A–D that are widely distributed throughout Asia. Using geographically weighted location averages to approximate each haplogroup centroid (red dots) and then buffering to form 4000 km interaction spheres (black circles), a core area (bold ellipse) that probably served as both a genetic and cultural hearth for Native Americans is identified.

Using a geographical approach to explore modern mtDNA distributions yields some intriguing insights into the distant past of the NEG and the Americas (Gillam *et al*., 2016). Native American mtDNA haplogroups are known to be dominated by groups A–D that are widely distributed throughout Asia, including the NEG (McDonald, 2005). By digitizing their distributions using a GIS system, it is possible to derive geographically weighted average estimates of their central locations (Figure 2). From north to south, Haplogroup C centres east of Skovorodino, Russia, at 54° north latitude and 125° east longitude. Haplogroup D falls near Shenyang, China, at 42° north latitude and 124° east longitude. Haplogroup A centres west of Shanghai at 32° north latitude and 119° east longitude. Finally, the southernmost Haplogroup B falls north of Hong Kong, at 26° north latitude and 114° east longitude. The maximum distance between these centres is approximately 4000 km, which can in turn be used as a hypothetical geographical range or interaction sphere for each haplogroup's occurrence in prehistory. These interaction spheres do not represent individual band or macroband territories, but rather the potential range of each haplogroup's wider population extent owing to exogamy. By buffering that distance from each centre, the overlap of these forms an elliptically shaped ‘core area’ where haplogroups A–D most likely intermingled, that likewise overlaps the NEG during the LGM. This core area ranges from the Altai Mountains and Lake Baikal in the northwest, southeastward through the Amur basin and northeastern China, ending on the Pacific coastlines of the Archipelago. Thus, the NEG probably played a major role in the cultural trajectories of humanity throughout East Asia and into the Americas during the Late Pleistocene, particularly as it relates to the ebb-and-flow movement of populations prompted by stadial and interstadial climactic episodes and related cultural adaptations and interactions through time.

As previously mentioned, North and South American genomic data are beginning to shed some light on the problem as well. Recent genetic evidence from the Anzick-1 burial in Montana (Rasmussen *et al*., 2014) revealed the founding mtDNA haplogroup, D4h3a, supporting a coastal migration, and the Y-chromosome haplogroup Q-L54*(xM3) that is known only from southern Siberian populations (Battaglia *et al.*, 2013), thus providing a potential empirical link to the Archipelago (cf. Kivisild *et al*., 2002). The Paisley Cave coprolites in Oregon also tie founding mtDNA haplogroups A2 and B2 to Far East Asia, originating around 14,000 and 16,500 BP, respectively (Gilbert *et al.*, 2008). Likewise, NRY analyses recently revealed a link between Central and South American Y-chromosome haplogroup Q-L54*(xM3) and Kamchatka in the Russian Far East (Battaglia *et al.*, 2013), a likely step along the coastal trail from the NEG to the Americas.

Genomic evidence also suggests that Native American ancestors were genetically isolated prior to migrating into mainland North and South America (Forster *et al.*, 1996). The mtDNA analyses suggest that genetic isolation occurred during the LGM from ca. 23,000 to 19,000 BP, followed by a rapid population expansion from 18,000 to 15,000 BP (Fagundes *et al.*, 2008). Arguably, the most likely region in East Asia with an archaeological record to support these mtDNA analytical results is the Archipelago. The date ranges for population isolation, expansion and migration closely correlate with technological and cultural factors present in the archaeological record there at the time.

Likewise, recent genome-wide single-nucleotide polymorphism studies revealed that, unlike all other East Asian populations, the Ainu of northern Japan and Sakhalin Island, Russia, have a closer genetic association with northeastern Siberians than their central Siberian counterparts (Jeong *et al.*, 2016; Jinam *et al.*, 2012). An ancient genomic study on individuals from Devil's Gate Cave (~7700 BP) in the Russian Far East proved to be a good East Asian ancestry for the Saqqaq individual, although it may not represent the initial wave into the New World (Sikora *et al*., 2019). We propose here that genetic isolation and subsequent migration are far more likely to be from a large island/peninsular setting (i.e. the Archipelago) with low population density, diverse cultural adaptations and nearly 20,000 years longer occupation (from ca. 38,000 BP), than a continentally linked landmass (i.e. Beringia) with little archaeological evidence dating prior to 15,000 BP (cf. Forster, 2004). Beringia appears to have been occupied much too late to have been more than a bridging environ for the initial coastally adapted migrants around 16,000 BP, linking the Old and New Worlds of the terminal Pleistocene until the subsequent migration of interior/terrestrially adapted central Siberians soon thereafter (ca. 15,500 BP).

### Discussion

The NEG clearly influenced both the cultural trajectories and migrations of humanity throughout northern Eurasia and into the Americas. Initial migration and settlement of the NEG probably followed a northerly route from west to east by ca. 45,000 BP (Zwyns *et al.*, 2019), skirting the northern limits of the NEG at that time. In contrast, the peopling of the Americas probably followed the coasts from the more southern, coastal latitudes of the NEG by ca. 16,000 BP. The Native American haplogroups A–D coalesce in a core area within the NEG (Figure 2), stretching from Mongolia to the Japanese Archipelago, forming a cultural hearth along the coasts and riverine environs of the Pacific Rim at the eastern extreme of this core area. The NEG during the Late Pleistocene (ca. 45,000–12,000 BP) probably experienced radical shifts in the presence and absence of humanity over time. During cold stadial periods, populations moved southward to more favourable environs and returned during warmer interstadials when the environment moderated and game likewise returned.

This episodic pattern of Pleistocene migration in the NEG not only influenced the cultural dynamics and trajectories of the area, but also set the stage for the peopling of the Americas after 17,000 BP with the appearance of pottery and more sedentary hunter–gatherers in the Far East. Growing regional territoriality would have pressured the lifeway of traditional, highly mobile fisher–hunter–gatherers, i.e. it provided an incentive to migrate northeast along the coast towards the Americas, beyond merely having the ability to do so. The NEG of the Archipelago is therefore a particularly strong candidate as a Paleoamerican hearth.

## Historical linguistics: the origin of the Turkic family and its early dispersal via the NEG

Some of the key observations on the socio-cultural background of the NEG can be illustrated with a case study on the origin and early dispersal of one of the most widespread language families in Northern Eurasia, the Turkic family. While most of its representatives – including the most spoken languages, such as Turkish, Azeri, Uzbek, Kazakh and Uyghur – are currently distributed far from the NEG, primarily in the steppe and further south, the Turkic prehistory was equally connected to the NEG forests, as we show below. A third of the ca. 35 contemporary Turkic languages are still predominantly spoken within the NEG. Those include most of the Siberian Turkic languages, which dominated the taiga before the Russian expansion, as well as the northernmost Kipchak languages (Tatar, Bashkir and Karaim) and Chuvash (the only surviving member of Bulgharic, which is the most divergent branch of Turkic). Below we apply a set of historical linguistic techniques in order to infer the ecological and cultural background of the Proto-Turkic language and the early dispersal of the family via the NEG.

### The Proto-Turkic homeland

**Figure 3.**
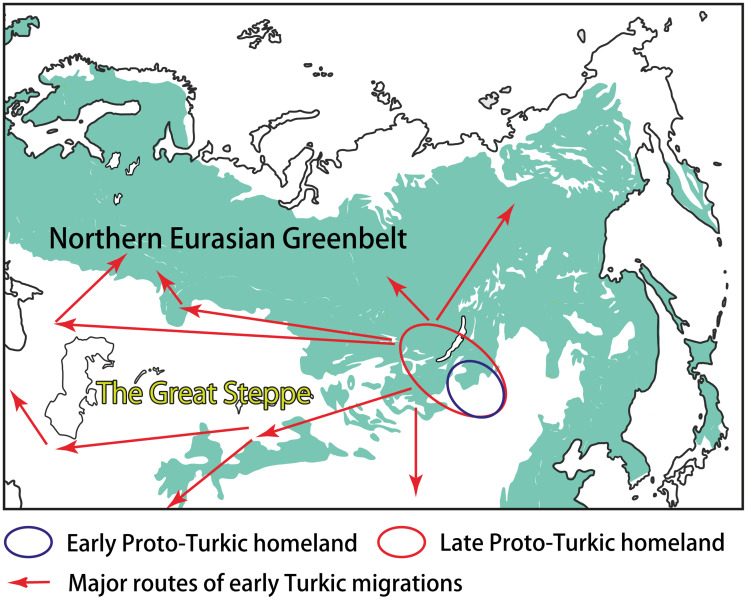
The homeland(s) and the early migrations of the Turkic peoples.

Most linguists and historians agree that Proto-Turkic, the common ancestor of all ancient and contemporary Turkic languages, must have been spoken somewhere in Central-East Asia (e.g. Róna-Tas, 1991, p. 35; Golden, 1992, pp. 124–127; Menges, 1995, pp. 16–19). Prior to the split of the proto-language at the end of the first millennium BCE, the Proto-Turkic speakers may have occupied the vast area stretching from the Sayan–Altai Mountains, South Siberia, in the west, to Eastern Inner Mongolia in the east. Such a wide distribution was probably the result of nomadic pastoralist expansions that started in the region ca. 1200 BCE, according to the recent findings (Taylor *et al.*, 2017, p. 50). The ultimate Proto-Turkic homeland may have been located in a more compact area, most likely in Eastern Mongolia, that is, close to the ultimate Proto-Mongolic homeland in Southern Manchuria and the ultimate Proto-Tungusic homeland in the present-day borderlands of China, Russia and North Korea (Janhunen, 2010, pp. 293–294; Robbeets *et al.*, in press; Figure 3). This hypothesis would explain the tight connections of Proto-Turkic with Proto-Mongolic and Proto-Tungusic, regardless of whether one interprets the numerous similarities between the three Altaic families as partly inherited or obtained owing to long-lasting contact.

Recent DNA studies show that starting from the end of the second millennium BCE, the East Asian-related components were already found in numerous populations in Central Asia and Eastern Europe (Narasimhan *et al.*, 2019). By the Iron Age, populations (e.g. Xiongnu) with primarily East Asian ancestry moved westward on a large scale, which combined in different proportions with local populations who were originally Indo-European speakers with largely west Eurasian ancestry that shifted their languages to Turkic (Damgaard *et al.*, 2018). Modern DNA of multiple Turkic populations showed that the Turkic peoples shared their ancestry with populations from southern Siberia and Mongolia, supporting the hypothesis that they originated there (Yunusbayev *et al.*, 2015; Tambets *et al*. 2018). Although current genetic evidence is not adequate to track the exact time and location for the origin of the proto-Turkic language, it is clear that it probably originated somewhere in northeastern Asia given the fact that the nomadic groups, such as the Rouran, Xiongnu and the Xianbei, all share a substratum genetic ancestry that falls into or close to the northeast Asian gene pool (Ning et al., in press; Li *et al.*, 2018).

The Proto-Turkic habitat can be viewed as an ecological junction area on the boundary of the NEG and the steppe. Linguistically, this diversity is mirrored in the contact history of Proto-Turkic, which included interactions with both the languages of the steppe, such as East Iranian and Tocharian, and the languages that are associated with the northern forest zone, such as Samoyedic and Yeniseian (Dybo, 2007, pp. 115–169). Additionally, the complex ecological and cultural background of Proto-Turkic can be inferred by means of Palaeolinguistic reconstruction, an approach implying strong correlation between the reconstructability of certain terms in a protolanguage and the familiarity of its speakers with the artefacts and phenomena which those terms denoted (see, e.g. Robbeets, 2017a, pp. 9–12, including a discussion on the general applicability of this method, as well as its challenges and limitations).

### Proto-Turkic vocabulary of the natural and cultural environments

The terms that refer to the natural environment and are reconstructable to the Proto-Turkic stage indicate that the earliest habitat of the Turks was home to diverse ecosystems, landscapes and species. While a common misconception links the Proto-Turkic homeland to the steppe environment only, a crucial role of the NEG for (Late) Proto-Turkic speakers is emphasized by reconstructed lexemes denoting different types of forest landscapes, as well as numerous terms for forest flora and fauna.

Proto-Turkic lexemes referring to forest landscapes include such terms as *orman ‘forest’, *jïɬ ‘mountain forest’ and *bük ‘thicket’. There are numerous reconstructed terms for broad-leaf tree species, including *kadïŋ ‘birch’, *tōř ‘birch bark’, *tal ‘willow’, *jöke ‘lime-tree’, *tẹrek(ə) ‘poplar’ and *kebrüč ‘ash-tree’. Terms for coniferous trees are rather scarce (e.g. *kadï ‘pine’, *tï̄t ‘larch-tree’) and their Proto-Turkic status is not universally accepted. Helimski (2000, pp. 301–303), following Róna-Tas (1988, pp. 745–746), explains the two terms for coniferous species as borrowed from Proto-Samoyedic, *kaəti̮ə ‘spruce’ and *ti̮te̮ŋ ‘Siberian pine’, correspondingly associating this contact event with the northward expansion of the early Turkic speakers from Mongolia into the Siberian taiga. Notably, Dybo (2007, pp. 135–137) argues against the borrowed nature of these Proto-Turkic terms, implying that coniferous forests may have been a part of the Proto-Turkic habitat.

Terms for forest fauna that can be reconstructed to Proto-Turkic include *bulan ‘elk’, *sïgun ‘red deer’, *adïg ‘bear’, *kīɬ ‘sable’, *tẹgiŋ ‘squirrel’ and *kunduř ‘beaver’. While a tentative eastern Uralic, i.e. Samoeydic or Ugric, origin was discussed for some of the Turkic forest fauna terms (e.g. Räsänen, 1969, p. 470), Dybo (2007, pp. 172–176) rejects all such proposals except the case of *kunduř < Proto-Ugric *kuntз-l’ ‘beaver’ (most likely borrowed as a trade term).

A steppe component in the Proto-Turkic natural environment is highlighted by such reconstructed terms as *jařï ‘steppe’, *bōr(ə) ‘infertile land’ and *kum ‘sandy land’ as well as terms for steppe fauna: *karsak ‘steppe fox’ and *jumran ‘ground squirrel’. There are numerous lexemes referring to mountain landscapes, e.g. *tāg ‘mountain’, *kïř ‘highland’, *kaja ‘cliff, rock’ and *korum ‘stone river’. Terms for lowland landscapes are far less abundant (e.g. *si̯ař ‘marsh, swamp’).

The Proto-Turkic homeland must have been rich with water resources, as is evident from such reconstructed terms as *jul ‘stream’, *ügüř ‘small river’, *örs(en) ‘river’, *etül ‘big river’, *köl ‘lake’. Curiously, a term for a large body of water (sea?) was known to the speakers of Proto-Turkic (*teŋiř). This may be another indication that the original habitat of the Turks was located not too far from the seashore, although there is otherwise no maritime vocabulary in Proto-Turkic. Terms for freshwater fish species include, e.g., *si̯āřan ~ *si̯āřukan ‘common carp’, *čapak(ə) ‘a kind of cyprinid (bream or roach)’, *jājïn ‘catfish’ and *čortan ‘pike’. The terms for water birds that can be reconstructed to Proto-Turkic are *kāř ‘goose’, *kogu ‘swan’, *ebürdek ‘duck’ and *aŋït ~ *aŋïř ‘a kind of duck’.

The complex natural environment may account for the diverse cultural background of the Proto-Turkic-speaking community. The Proto-Turkic subsistence strategy included an agricultural component, a tradition that may have been inherited from the earlier Proto-Altaic stage and ultimately went back to the origin of millet agriculture in Northeast China (Robbeets, 2017b; Savelyev, 2017). The agricultural vocabulary reconstructed to Proto-Turkic includes terms for cultivated cereals (*ügür ‘broomcorn millet’, *arba ‘barley’ and *budgaj ‘wheat’), bread production (*i̯unk ‘flour’), farming techniques (*tarï- ‘to cultivate land’, *ek- ‘to sow’, *or- ‘to reap’ and *sabur- ‘to winnow grain’) and tools (*kerki ‘a type of mattock’ and *ek-eg ‘plough’).

A nomadic, pastoralist lifestyle reached the eastern steppe by the end of the second millennium BCE (Taylor *et al.*, 2017; Janz *et al.*, 2017), and it became the basis of the Late Proto-Turkic subsistence in the first millennium BCE. Consequently, the Proto-Turkic language has developed extensive nomadic pastoralist vocabulary, including terms for domestic animals (e.g. *sïgïr ‘cattle’, *toklï ‘lamb’, *adgïr ‘stallion’ and *kulum ‘foal’), horse-riding (*at ‘riding horse’ and *edŋer ‘saddle’) and dairy products (*ajran ‘a kind of salty yoghurt’ and *torak ‘a kind of cheese or quark’). According to some accounts (e.g. Dybo 2007, pp. 116–117), a number of Proto-Turkic pastoralist terms were borrowed from Indo-European and, specifically, Eastern Iranian (e.g. *tạ̄na ‘heifer’, cf. Khotan Saka *dīnū* ‘cow’ of Iranian origin).

Arguably, hunting, fishing and gathering remained relevant for the Proto-Turkic community as a subsistence strategy at all stages of its history. The reconstructed terms for hunting include such lexemes as *kejik ‘game’ (most likely, hunted ungulates), *āb ‘hunt; enclosing of game’ and *āg ‘(hunting) net’.

Other reconstructed components in the Proto-Turkic culture include handicraft (e.g. *ed- ‘to tan leather’ and *kidiř ‘felt’), metallurgy and warfare (*tẹmür ‘iron’, *kïlïč ‘sword’, *ok(ə) ‘arrow’ and *jagï ‘war’) and housing with terms referring to both mobile dwellings (*koč- ‘to migrate’, *jūrt ‘territory where nomads roam’ and *ōtag ‘temporary dwelling’) and stationary dwellings (*eb ‘house’, *ker-men ‘fortress’ and *bi̯alï-k ‘town’); for a more detailed overview, see Tenišev *et al.* (2001, pp. 356–579).

To sum up, the palaeolinguistic reconstruction points to a mixed subsistence strategy and complex economy of the Proto-Turkic-speaking community. It is likely that the subsistence of the Early Proto-Turkic speakers was based on a combination of hunting–gathering and agriculture, with a later shift to nomadic pastoralism as an economy basis, partly owing to the interaction of the Late Proto-Turkic groups with the Iranian-speaking herders of the Eastern Steppe. Within the reconstructed Proto-Turkic vocabulary of cultural environment, it is the pastoralist terminology that is most liable to contact phenomena. Another semantic domain featuring some reliably non-genuine items (e.g. loans from Old Chinese) is the terminology of metals (Tenišev *et al.*, 2001, pp. 401–413). However, for most of the Proto-Turkic cultural terms, no evident source of borrowing is known in the neighbouring languages; moreover, some cultural items in Proto-Turkic have plausible Altaic parallels (cf. Savelyev, 2017), thus implying a certain degree of cultural continuity with the preceding Proto-Altaic period.

### Early Turkic migrations and cultural transmission via the NEG

The further interplay of ecological and cultural factors as well as the role of the NEG in the early Turkic history can be outlined as follows. The Proto-Turkic language split off ca. 100 BCE (Mudrak, 2009, p. 181; Savelyev and Robbeets, 2020) when its speakers started to leave the ecological junction area on the eastern fringes of the NEG. Over the following one and a half millennia, the Turks re-settled across the vast expanses of Eurasia, partly as a result of southward and southwestward migrations, reaching as far as northwest China, Iran, the eastern Mediterranean and Asia Minor (Figure 3). On the other hand, some groups of the Turkic speakers moved deeper into the NEG area, coming into contact with local populations and adapting to the new ecological conditions and challenges for subsistence. The two most illustrative examples of such an interaction can be found in central-eastern Siberia – and far to the west, over the Ural Mountains, in the Volga–Kama basin.

The Bulghar tribes, who spoke the most divergent variety of Turkic, arrived from their East Asian homeland and settled in Europe during the Migration period between ca. the fourth and the sixth centuries CE. After the Great Bulgharia, a Bulghar polity in the plain areas of Ciscaucasia and the adjacent parts of the Pontic steppe, collapsed in the mid-seventh century, a group of the Bulghars moved northwards and reached the ecological border between the steppe and the NEG in the Volga–Kama region in the eighth century (Savelyev, in press). From that time, until quite recently, the speakers of Volga Bulghar and its descendant, the Chuvash language, were in permanent contact with the local Uralic populations, including the speakers of Mari, Permic and, to a lesser extent, Mordvin. Consequently, cultural terms of Bulgharic origin became extremely widespread in the Uralic languages of the region, encompassing almost all relevant semantic fields. This property of the Bulgharic–Uralic contact vocabulary reflects the process of cultural interaction in which the Turkic-speaking elite took the dominant role. One of the few domains where the flow of linguistic loans was in the opposite direction was the vocabulary referring to life in the northern forest (e.g. Chuvash *lə̂s* ‘fir-needle, conifer branch’ < Udmurt *li̮s* ibid., Chuvash *jəltər* ‘ski’ < Mari *jol* ‘foot’ + *ter* ‘sled’). The driving force behind the borrowing of such terms by the Bulgharic speakers was most likely the fact that the newcomers had to (re-)adapt their lifestyle to the NEG after many centuries spent in a steppe environment. Several centuries later, another group of southern migrants – the speakers of the northern Kipchak varieties, to become the modern Tatar and Bashkir languages – went through the same process of the incorporation of an NEG-based component into their lifestyle, which was mirrored in the adoption of specialized forest-related terms from Uralic.

In the Siberian taiga from the pre-Russian period, one might observe a similar pattern, whereas the Turkic speakers were in a socially prestigious position and ensured the flow of technologies and cultural concepts to the adjacent languages – and lexemes for those technologies and concepts in exchange for terms referring to the natural environment (substratum loans). During the first millennium CE and later, the Siberian Turkic speakers made their way into inner Siberia and interacted with the speakers of those language families whose habitat is more commonly associated with the NEG. Contacts of this type were particularly intensive in Southern Siberia, with borrowing scenarios involving such languages as Yeniseian and Samoeydic (e.g. Helimski, 1991), although they were a no less important factor in the cultural and linguistic history of the Turks of Northern Siberia, i.e. the Yakuts and the Dolgans (Pakendorf and Stapert, in press).

The genetic relations of Turkic-speaking populations with their geographically close non-Turkic neighbours have been well documented elsewhere (Rasmussen *et al.*, 2010; Behar *et al.*, 2010). Turkic populations, especially those from the Volga–Uralic region (Chuvashes, Tatars and Bashkirs), demonstrate a genetic profile that is maximized in the Siberian Uralic-speaking populations (e.g. Nganasans) (Yunusbayev *et al.*, 2015). Ancient DNA, however, also showed a wide spread of Siberia Nganasan-related ancestry among the Uralic-speaking populations in northern Europe, which was introduced into the region at least 3500 years ago (Lamnidis *et al.*, 2018), documenting a long-term potential gene flow between at least part of the Turkic- and Uralic-speaking populations.

## Discussion and conclusion

As demonstrated, the mtDNA geographically weighted average estimates reveal a high probability that the Native American mtDNA haplogroups originated in the southern NEG from the Sayan–Altai Mountains to the Japanese Archipelago. The results suggest that the climatic deterioration of the LGM during the Late Pleistocene could be a major driving force pushing interior populations to encroach upon coastal areas and/or populations, ultimately inducing the peopling of the Americas. On the other hand, the linguistic analysis of the Proto-Turkic language family from the first millennium BCE onward indicates that the family's homeland is a rather compact area in the southern fringe of the NEG in Northeast Asia. This region, near eastern Mongolia, contained diverse ecosystems that enabled hunting–fishing–gathering to remain important components of subsistence.

It should be noted that the estimated origin of the Native American mtDNA haplogroups and the presumed homeland of the Proto-Turkic language family largely overlap each other in the southeastern NEG from the Altai Mountains, via the Amur River Basin, to the Japan Sea coast. Likewise, this is the area where the earliest ceramic vessels developed and spread (Figure 1c). These facts raise the question of why the Altai–Amur–Japan area repeatedly became the starting point of major historical events, particularly those related to human migrations. Our interdisciplinary approach proposes a possible explanation from an ecological perspective. That is, it is because this area has historically included the diverse biomes of the NEG.

It is widely recognized that hunter–gatherer strategies require exploitation of a wide range of resources from different environs, this according to the expectations of optimal foraging theory that every society tries to guarantee the most benefit at the minimum cost (e.g. Smith, 1983). In particular, logistic hunter–gatherers, with a degree of sedentary lifestyle in the Holocene, are known to often set residential bases at eco-junctions, i.e. borderlines of different ecosystems, such as waterfronts between aquatic and land environments, so that they can obtain various resources throughout the year (Gillam *et al*., 2010; Uchiyama, 2015). Located along the boundary of the inland steppe, the Altai–Amur–Japan area includes various kinds of biomes, i.e. different kinds of forests, grasslands, and freshwater and maritime environments. Terrain, with a large variety of surface features, also provides environments with various kinds of biomes packed into relatively small regions. Such biological and geographical conditions allowed people to access diverse resources in different environments with minimum mobility, offering multiple eco-junctions as places to settle.

Areas containing varied eco-junctions must have had relatively high-level population densities and been places where social groups with different lifestyles could meet, enhancing potential to create new cultures or cultural traits. Besides the greater Altai–Amur–Japan area, there are several other areas with similar environmental conditions in the NEG, such as the Ural Mountains and the Volga–Kama Basin. These areas of the NEG may have likewise formed regional cultural cores. While various cultures were created in these core areas and their vicinities, archaeological and linguistic traces attest that they were connected to each other through long-distance geographical networks. On the other hand, once living environments deteriorated owing to growing population, climatic fluctuations or both, and such conditions may have served as a great incentive for people to move away from already highly populated NEG cultural cores into other areas, as seen in the peopling of North America and the split and expansion of the Proto-Turkic language family. The NEG should be viewed as one united socio-cultural system that played a vital role in the processes of cultural formation and migration across Eurasia and beyond.

## Data Availability

All data used for this article can be found in the published literature cited in the references.
